# Motivational climate, self-determination, burnout, and mindfulness in adolescent football players from a professional academy in virtual settings

**DOI:** 10.3389/fpsyg.2025.1732005

**Published:** 2026-01-08

**Authors:** Mario Reyes-Bossio, Natalia Veran-Casanova, Franco Ascenzo-Bravo de Rueda, Andy Sánchez-Villena, Mariel Delgado-Campusano, Veronica Tutte-Vallarino, Regina Brandão

**Affiliations:** 1 Facultad de Psicología, Universidad Peruana de Ciencias Aplicadas, Lima, Peru; 2 Sociedad Iberoamericana de Psicología del Deporte, Santiago de Compostela, Spain; 3 Vicerrectorado de Investigación, Universidad Señor de Sipán, Chiclayo, Peru; 4 Universidad Católica del Uruguay, Montevideo, Uruguay; 5 Universidade Sao Judas Tadeu, São Paulo, Brazil

**Keywords:** adolescent football (soccer), athlete burnout, empowering and disempowering coaching, mindfulness, motivational climate, network analysis, self-determined motivation, virtual training

## Abstract

**Background:**

The COVID-19 pandemic shifted sports training to virtual formats, impacting athletes’ motivation, well-being, and mental health. In this context, motivational climate, self-determined motivation, mindfulness, and burnout are key factors for understanding adolescents’ psychological adjustment in football.

**Methods:**

This study employed a cross-sectional design, with all variables collected at a single time point during mandatory virtual training. This cross-sectional study surveyed 154 adolescent football players (M = 15.9 years) from a Peruvian professional academy during mandatory virtual training. Participants completed the EDMCQ-C, SMS, MAAS-5, and ABQ. A psychological network analysis was performed in R using non-regularized partial correlations and bootstrapped stability estimates.

**Results:**

An empowering climate was positively associated with intrinsic motivation and mindfulness, whereas a disempowering climate was linked to extrinsic motivation and burnout. Extrinsic motivation emerged as the most central node in the network, and mindfulness functioned as a bridging node that buffered the spread of demotivation toward exhaustion. The model showed adequate stability (CS = 0.44).

**Conclusion:**

Empowering motivational climates and mindfulness protect adolescents’ psychological wellbeing, whereas controlling coaching and extrinsic motivation heighten the risk of burnout. These findings support incorporating autonomy-supportive coaching and brief mindfulness practices in youth sport training and coach education programs.

## 1 Introduction

The COVID-19 pandemic brought an unprecedented transformation to youth sport. The closure of facilities and suspension of in-person sessions forced training to move online, profoundly reshaping coach–athlete interaction, motivation, and psychological wellbeing (Hussain et al., 2023; Zhao et al., 2022). In Peru, as in many other countries, professional football academies adopted remote systems with synchronous and asynchronous sessions led by coaches seeking to sustain motivation, adherence, and physical preparation despite restrictions. This context particularly affected adolescents, who experienced disrupted routines, reduced social interaction, lower perceived competence, and heightened physical and psychological stress (Dauty et al., 2021; Philippi et al., 2024).

The shift to virtual training during the pandemic generated particularly critical technical and physical limitations in high-performance sports, where movement specificity and immediate feedback are fundamental. Several studies show that athletes reduced the load, intensity, and technical variability of their home training due to a lack of resources and difficulties in replicating the stimuli of in-person practice (Purc-Stephenson et al., 2022). In professional soccer, virtual platforms diminished the quality of technical and tactical feedback and social interaction among coaches, players, and families (Maurice et al., 2021). Similarly, coaches of team sports reported difficulties in maintaining individualized motor correction and group dynamics in an online format (Teodorescu et al., 2021). Overall, the evidence indicates that virtual training does not functionally replace in-person training and can negatively impact the athlete’s physical progression, technical accuracy, and motivational experience.

International studies confirmed that health restrictions increased the risk of exhaustion, anxiety, and reduced wellbeing (Sokić et al., 2021; Uroh and Adewunmi, 2021), alongside more stress and symptoms of psychological fatigue among young athletes (Samuel et al., 2022). This panorama highlighted the need to strengthen psychological resources such as autonomy, emotion regulation, and resilience. Football, given its high physical and emotional demands, made especially evident how confinement conditions undermined motivation, energy, and sense of achievement in youth.

Analyzing the psychological factors underpinning performance and wellbeing in virtual settings is essential both scientifically and practically. The joint consideration of Empowering Coaching, Self-Determination Theory, mindfulness, and the sport burnout model allows us to understand dynamic relations among motivation, emotions, and strain. Practically, such evidence can guide psychological and pedagogical interventions (Reyes-Bossio et al., 2022a) that promote motivation and prevent burnout in youth academies.

Psychological network analysis provides an innovative approach, enabling visualization of interactions among variables and identification of central nodes or influential factors (Epskamp and Fried, 2018). In Latin American contexts, applying this method with youth athletes can generate locally grounded evidence and offer contextualized strategies for wellbeing and sustainable participation.

The Empowering/Disempowering Coaching model (Duda and Appleton, 2016) posits that coaches’ behaviors shape an interpersonal context that can stimulate or inhibit athletes’ motivation and wellbeing. Empowering climates are marked by autonomy support, clear structure, and positive social involvement conditions that satisfy basic psychological needs and foster more self-determined motivation. In contrast, disempowering climates are characterized by controlling behaviors, punishment, excessive pressure, and negative feedback, which frustrate those needs and generate distress.

Recent evidence shows that empowering environments predict greater enjoyment, persistence, mental health, and resilience, whereas disempowering contexts are linked to stress, anxiety, exhaustion, and burnout symptoms (Appleton et al., 2023; Castillo-Jiménez et al., 2022). Coach-education initiatives grounded in Empowering Coaching have yielded meaningful improvements in creating healthier motivational climates, helping instructors learn to cultivate dedication, effort, and genuine engagement among young athletes (Castillo et al., 2015). A paradigmatic example is the European PAPA project (Promoting Adolescent Physical Activity), which showed that empowering-oriented training enhances self-determined motivation, enjoyment, and adherence.

Over the last two decades, multiple studies have underlined that the coach-created motivational climate is a key determinant of commitment and sport responsibility, as it promotes positive emotions (pleasure, wellbeing, enjoyment) together with a healthy performance orientation toward personal improvement (Castillo et al., 2015; Gutiérrez-García et al., 2019). This effect is sustained by the coach’s expertise and the surrounding environment’s capacity to generate meaning and passion for sport (Herrera et al., 2025; Reyes, 2009). Accordingly, coaches who convey an empowering atmosphere centered on trust, cooperation, and autonomy facilitate adaptive psychological subdimensions such as intrinsic motivation, enjoyment, and perceived progress. Conversely, disempowering styles reinforce dysfunctional dynamics (mental/physical fatigue, excessive rivalry, dropout) through pressure, intimidation, or constant criticism, prioritizing results, using threats as control, and emphasizing mistakes as punishment (Castillo et al., 2015; Gutiérrez-García et al., 2019; Moreno et al., 2019; Moulds, 2023).

In youth football, several studies confirm the direct impact of the motivational climate on players’ mental wellbeing. Castillo-Jiménez et al. (2022) found that a disempowering climate relates to stronger dropout intentions and frustration of basic psychological needs, whereas an empowering climate fosters self-determined motivation and sport continuation. Complementarily, Martínez-González et al. (2025) reported that players perceiving more adaptive climates high empowering and low controlling show greater vitality, need satisfaction, and favorable motivational regulation. Converging evidence from Brandão et al. (2021) and Birr et al. (2023) across football, rugby, and basketball indicates that perceived disempowering behaviors are associated with emotional fatigue and stress/burnout symptoms, while empowering climates facilitate better adjustment and less distress. Overall, this evidence underscores the importance of preparing coaches to construct empowering climates in both in person and virtual contexts to enhance performance and protect athletes’ mental health.

Self-Determination Theory (Ryan and Deci, 2017) conceptualizes human motivation along a continuum of self-determination, from intrinsic motivation (engaging for pleasure, enjoyment, or learning) to extrinsic motivation (guided by rewards, pressures, or external expectations), culminating in amotivation (absence of intention or meaning). The degree of self-determination depends on the satisfaction of three basic psychological needs: autonomy (acting with a sense of volition), competence (feeling effective and capable), and relatedness (feeling connected and valued by others).

In youth football, SDT provides a robust framework for understanding motivation. In a longitudinal study with elite players, Hendry et al. (2019) showed that self-determined motivation fluctuates across the season as a function of age, expertise, and the training-to-competition ratio, concluding that satisfying autonomy and competence is essential to sustain enjoyment and persistence. Complementarily, Monteiro et al. (2018) verified a motivational sequence in which a task-involving climate and satisfaction of basic needs predict self-determined motivation and perceived effort, indicating that coach-provided autonomy and competence support increases commitment. Extending to team sports, Amado et al. (2019) found that higher self-determined motivation is associated with better self-talk and greater team potency, reinforcing the relevance of a supportive social environment.

During the COVID-19 pandemic, reduced social contact and diminished direct coach support particularly affected relatedness, heightening motivational vulnerability (Sokić et al., 2021). In this scenario, promoting autonomy through self-referenced goals and strengthening competence via positive feedback and calibrated challenges are effective strategies to mitigate adverse psychological effects of virtual training and sustain engagement under isolation.

Mindfulness is understood as the capacity to deliberately and consciously direct attention to present-moment experiences, accepting them without judgment (Quaglia et al., 2015). While conceptually rooted in Buddhist contemplative traditions (Desbordes et al., 2015), related practices of awareness and presence also appear in other spiritual streams. In recent decades, the construct has been integrated into Western contexts with a secular, scientific focus as a strategy for emotion regulation and psychological wellbeing (Bilevicius et al., 2018), and has gained prominence in sport psychology given its potential to enhance regulation and attentional control. Evidence indicates that mindfulness reduces stress, anxiety, and athlete burnout while improving wellbeing (O’Connor et al., 2022; Trujillo-Torrealva and Reyes-Bossio, 2019).

In football-related settings, mindfulness shows promising effects on emotion regulation and attentional performance. Oguntuase and Sun (2022) implemented a mindfulness program with elite footballers and found improvements in resilience, self-confidence, and emotional regulation. In collegiate football, Wu and Ai (2025) demonstrated that a brief mindfulness intervention improves visual control and attentional stability during pressured penalty kicks. A broader systematic review across multiple sports reported that higher mindfulness is associated with fewer burnout symptoms and better coping strategies (Li et al., 2019). In competitive football, Solmaz and Yarayan (2025) suggested that mindfulness mitigates the linkage between threat appraisal and negative emotions among elite players, implying a moderating role under stress. Finally, O’Connor et al. (2022) observed that dispositional mindfulness protected athletes from pandemic-related distress. In contexts such as Peru, brief mindfulness techniques embedded in virtual sessions could strengthen resilience and focus among youth players.

Sport burnout is conceptualized as a state of physical and emotional exhaustion, devaluation of participation, and reduced sense of accomplishment (Raedeke and Smith, 2001). It emerges when sport demands exceed athletes’ psychological and physical resources. Recent reviews confirm associations between burnout and disempowering climates, thwarted autonomy, and low social support (Woods et al., 2022; Zhao et al., 2022).

Among high-performance athletes, burnout has been linked to an imbalance between effort and psychological return across individual and team sports. Recent work with elite samples indicates that burnout symptoms are associated with discrepancies between sport investment and perceived gratification (Hilpisch et al., 2024). In youth sport, high psychological and physical demands predispose athletes to burnout in competitive settings (Malán-Ernst et al., 2025; Tutte-Vallarino et al., 2022; Woods et al., 2022). During the pandemic, the inability to compete and uncertainty about sport continuity increased risks of exhaustion and demotivation (Hussain et al., 2023). Combining Empowering Coaching with mindfulness-based interventions offers a promising strategy to reduce burnout and enhance emotional self-regulation.

The theoretical models underpinning this study can be understood as parts of the same motivational and self-regulatory system. The coach’s motivational climate influences the satisfaction or frustration of the basic psychological needs of Self-Determination Theory, such that task-oriented and autonomy-supportive climates are associated with greater wellbeing and less distress in team sports (Reinboth and Duda, 2006). In turn, the satisfaction of these needs fosters more self-determined motivation, which protects against burnout and enhances sports engagement (De Francisco et al., 2020).

Within this framework, mindfulness is conceptualized as a self-regulatory resource that helps manage competitive demands and has been negatively associated with burnout in athletes, both in meta-analytic reviews and in studies with elite athletes (Li et al., 2019; Zhang et al., 2016). The empowering training climate proposal explicitly integrates the contributions of Self-Determination Theory and Achievement Goal Theory, emphasizing that coach behaviors can be simultaneously more or less empowering or controlling, with clear implications for athletes’ motivation and mental health (Duda and Appleton, 2016). From this perspective, psychological network analysis offers a suitable tool for studying in an integrated way how these variables are organized in complex systems, identifying central nodes and patterns of interconnection between motivational climate, motivational regulation, mindfulness, and burnout (Epskamp et al., 2018).

Empirical studies in recent years have addressed the interplay among motivational climate, motivation, and wellbeing in young athletes. Appleton et al. (2023) showed that empowering climates buffer the negative effects of disempowering behaviors on psychological health. Castillo-Jiménez et al. (2022) reported that empowering climates predict satisfaction of basic psychological needs and intention to continue in sport. Li et al. (2019) demonstrated mindfulness as a protective factor against burnout during confinement, while O’Connor et al. (2022) found that dispositional mindfulness lowers psychological distress in high-performance athletes.

Recent work also highlights resilience and social support as central to adaptation. Hussain et al. (2023) documented that psychological resilience helped mitigate lockdown effects in international athletes, and Zhao et al. (2022) found that perceived social support mediated the relationship between burnout and mental health in youth footballers. Altogether, this evidence reinforces the need to promote positive motivational climates and emotion-regulation strategies in Latin American academies.

Despite the growing number of studies on the psychological effects of the pandemic on youth athletes, there is still no evidence that simultaneously integrates motivational climate, self-determined motivation, mindfulness, and burnout in virtual training contexts. Most research has focused on the general consequences of lockdown (Bates et al., 2021) or the physical and technical impact of remote training (Kalinowski et al., 2023) but has not addressed how these psychological processes interact under digital conditions. Similarly, although the adaptation of players, coaches, and families to online training has been described (Maurice et al., 2021), no previous study has applied psychological network analysis to model dynamic connections between these variables in adolescent soccer players, leaving a clear methodological gap. Therefore, this study advances the field by examining not only the magnitude of each construct but also the systemic structure of their interrelationships in a relatively unexplored context of sports virtualization.

In sum, motivational and emotional processes take on particular relevance when sport unfolds in non-face-to-face environments. In Peruvian youth football, virtual training during the pandemic posed an unprecedented challenge to maintaining motivation, group cohesion, and wellbeing. Examining how the coach-created motivational climate interacts with self-determined motivation, mindfulness, and burnout in this context clarifies the mechanisms that facilitate adaptation and commitment under isolation and uncertainty. Such knowledge is essential not only for strengthening the theoretical base of sport psychology in digital settings but also for guiding psychological support and instructional strategies that optimize learning and training in future emergencies or hybrid modalities—ultimately promoting more autonomous, resilient, and emotionally sustainable practice environments when physical contact and in-person sessions are constrained.

For this study, the psychological network approach was adopted, a method that models psychological processes as dynamic systems of interconnected elements. Within this framework, each variable is represented as a ‘node,’ and the connections between them reflect partial statistical dependencies within the system (Borsboom and Cramer, 2013). A ‘central node’ is the one that exerts the greatest structural influence on the rest of the network according to centrality indices, especially Expected Influence (EI), which allows for the identification of key psychological mechanisms for intervention (Opsahl et al., 2010). This approach is particularly useful in virtual contexts, where the interaction between motivational climate, motivation, mindfulness, and burnout can be reorganized differently than in face-to-face environments.

Aligned with this purpose, the present study aimed to analyze the interactions among motivational climate (empowering and disempowering), self-determined motivation, mindfulness, and burnout in adolescent footballers from a Peruvian professional academy during the pandemic period, using a psychological network analysis approach. Specifically, we sought to examine associations among the main psychological variables, identify the most central nodes within the motivational network, and explore the moderating role of mindfulness in the relationship between motivational climate and burnout indicators. We also evaluated the network’s stability and accuracy to provide robust empirical evidence informing psychological mechanisms of motivation and wellbeing in virtual training contexts.

## 2 Methods

### 2.1 Participants

The sample comprised 154 adolescent footballers (66.9% boys; 33.1% girls) engaged in synchronous and asynchronous virtual training; mean age M = 15.94 (SD = 3.18). Most were school students (76%); a smaller group were university students (7.8%) or not studying at the time (3.2%).

By sport category, boys were mainly from the 2006 (17.5%), 2004 (14.3%), and 2005 (13.6%) cohorts, whereas girls showed a balanced distribution across women’s professional, U-14, and U-16 teams (11% each). Regarding housing, most lived in homes of 70–120 m^2^ (52.6%), and 46.1% reported access to open spaces (garden/terrace).

Concerning physical condition during the pandemic, 40.3% reported only occasional declines, whereas 34% indicated frequent or continuous impact suggesting a moderate deterioration. Weekly activity varied: 27.3% trained 8–10 h, 19.5% trained 5–7 h, and 16.2% trained 14–16 h. Over three quarters remained active in virtual training (50.6% always, 27.3% most of the time), although only 19.5% always had adequate equipment (32.5% most of the time). Organization and technical follow-up were relatively high: 42.9% reported always being organized for training, and 50.6% reported constant coach monitoring.

To reinforce methodological transparency, the study applied the following inclusion criteria: (a) being an active member of the professional academy at the time of data collection; (b) participating in the virtual training sessions as scheduled by the academy; and (d) providing informed consent from parents or legal guardians, in addition to the athlete’s assent.

The exclusion criteria were: (a) having injuries or medical conditions that limited regular participation; (b) not attending the virtual training sessions during the data collection period; and (c) submitting incomplete data on the questionnaire.

Participants were recruited through an institutional announcement disseminated by the academy’s technical staff, inviting all eligible athletes to voluntarily participate in the study. It is worth noting that no athlete who met the inclusion criteria declined to participate.

### 2.2 Instruments

Motivational Climate Questionnaire–Coach (EDMCQ-C): Perceptions of the coach-created climate were assessed with the Empowering and Disempowering Motivational Climate Questionnaire–Coach (EDMCQ-C) (Appleton et al., 2016). The 34-item instrument comprises two global dimensions: Empowering Climate (17 items) autonomy support, task involvement, and social support and Disempowering Climate (17 items) controlling behaviors and ego-involving tendencies. Responses are recorded on a 1 (strongly disagree) to 5 (strongly agree) Likert scale. Previous studies report adequate factorial validity and internal consistency (*α* = 0.87 for Empowering; α = 0.85 for Disempowering) and cross-cultural invariance in youth sport (Appleton et al., 2023). We used the adolescent Spanish-language adaptation, preserving the bidimensional structure. For this research, the internal consistency values were adequate, with the following reliability values observed: *ω* = 0.92 for empowerment, ω = 0.90 for disempowerment.

Sport Motivation Scale (SMS): Motivation was measured using the Sport Motivation Scale adapted to Spanish by Balaguer et al. (2007). The SMS comprises 28 items across seven subscales: three intrinsic motivation facets (to know, to accomplish, to experience stimulation), three extrinsic regulations (identified, introjected, external), and amotivation. Responses use a 1 (does not describe me at all) to 7 (completely describes me) Likert scale. The Spanish version shows adequate internal consistency (*α* > 0.70) and factorial/theoretical validity in youth sport samples. For this research, the internal consistency values were adequate, with the following reliability values observed: *ω* = 0.89 for intrinsic motivation, ω = 0.91 for extrinsic motivation, and ω = 0.72 for amotivation.

Mindful Attention Awareness Scale (MAAS-5): Mindfulness was assessed with the five-item MAAS-5, validated in Spanish (Caycho-Rodríguez et al., 2019; Reyes-Bossio et al., 2022b). It measures the general disposition to attend to present-moment experiences consciously, using a 1 (almost never) to 6 (almost always) scale. The instrument is unidimensional and reliable (ω = 0.83, 95% CI [0.79, 0.85]) with adequate corrected item total correlations; its brevity favors field use with adolescents in sport settings. For this research, the internal consistency values were adequate, with the following reliability values observed: ω = 0.73 for mindfulness.

Athlete Burnout Questionnaire (ABQ): Burnout was measured with the ABQ (Raedeke and Smith, 2001), a 15-item instrument covering Physical/Emotional Exhaustion, Reduced Sense of Accomplishment, and Sport Devaluation. Responses range from 1 (never) to 5 (always). The ABQ shows strong internal consistency (total α ≈ 0.90) and construct validity across disciplines; we used the Spanish translation/adaptation by Arce et al. (2012). For this research, the following reliability values were observed, adequate for: ω = 0.73 for Physical/Emotional Exhaustion, and relatively low but acceptable (Katz, 2006) for Reduced Sense of Accomplishment (ω = 0.65) and for Sport Devaluation (ω = 0.65).

### 2.3 Procedure

The study was conducted during COVID-19 lockdown in coordination with a Peruvian professional academy that implemented synchronous and asynchronous virtual training. First, we contacted the technical management and coaching staff, explained aims, confidentiality, and administration procedures, and obtained institutional authorization. Parental or guardian consent and athletes’ assent were secured in line with the Declaration of Helsinki and local ethical standards. The study was approved by the UPC Faculty of Health and Psychology Research Ethics Sub-Committee (CEI 253–07-20 PI169-20).

Data were collected via a self-administered Google Forms questionnaire including sociodemographics (age, sex, category, education, training frequency, contextual conditions) and the EDMCQ-C, SMS, MAAS-5, and ABQ. The online assessment was completed during a scheduled virtual training session; completion time was ~20–25 min. Data were downloaded to CSV for cleaning and statistical analysis.

### 2.4 Data analysis

We computed descriptive statistics (mean, SD, kurtosis, skewness); values exceeding |1.5| were treated as evidence of non-normality (Pérez and Medrano, 2010). Network analysis was performed in R with bootnet, qgraph, and igraph. Networks were estimated using non-regularized partial correlations via ggmModSelect (Epskamp and Fried, 2018) and Spearman matrices due to non-normal distributions (Isvoranu and Epskamp, 2023). Node centrality was examined using Expected Influence, which is more suitable for networks with both positive and negative edges (Robinaugh et al., 2016). Network stability and accuracy were assessed with 1,000 bootstrap resamples (Epskamp, 2020); the stability cutoff followed the recommended CS > 0.25 (Epskamp et al., 2018).

## 3 Results

### 3.1 Descriptive statistics

Descriptive indices (Table 1) showed higher scores for empowering (M = 73.40; SD = 8.37), intrinsic motivation (M = 73.40; SD = 8.37), and extrinsic motivation (M = 60.40; SD = 14.80). Kurtosis and skewness exceeded ±1.5, indicating non-normality.

**Table 1:** Descriptive statistics.

Variable	Mean	Estándar Deviation	Skewness	Kurtosis
Mindfulness	21.20	4.98	−0.15	2.39
Empowering	73.40	8.37	−0.83	4.92
Disempowering	39.10	10.20	0.65	4.60
Intrinsic	73.40	9.06	−1.32	5.11
Extrinsic	60.40	14.80	−0.76	3.22
NoMotivation	9.15	4.71	0.76	2.70
Exhaustion	8.36	2.65	1.43	6.95
Decrease	12.60	2.58	1.14	5.15
Devaluation	8.16	3.30	1.28	4.31

### 3.2 Network analysis

**Figure 1:**
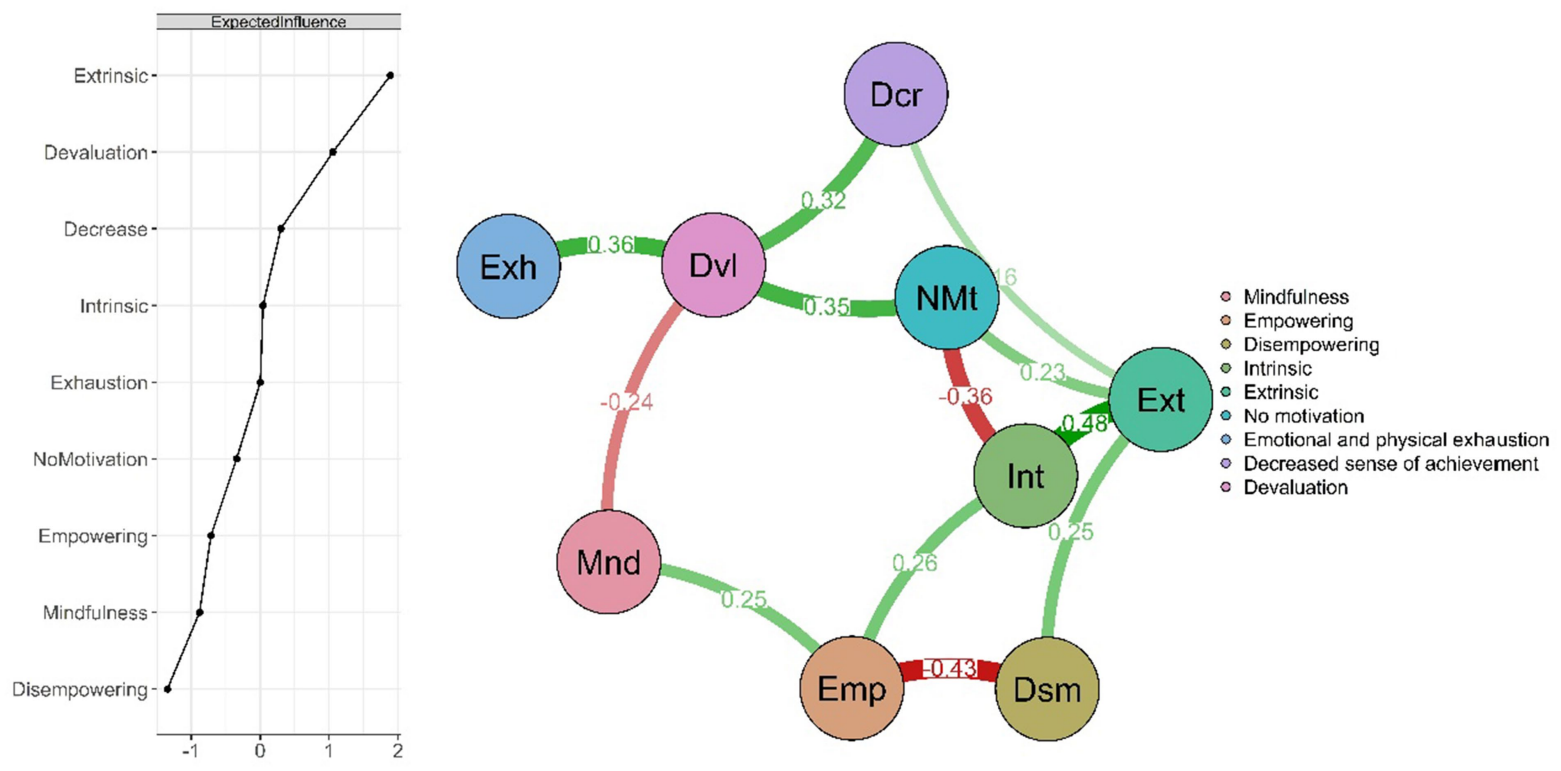
Network analysis.

Figure 1 displays the network results, based on correlations between the variables of motivational climate, motivation, burnout, and mindfulness. The nodes represent each subscale evaluated, while the edges indicate partial associations, controlling for the effect of the other variables in the system. The thickness and color intensity of each edge reflect the strength of the association, with green representing positive connections and red representing negative ones. With strong correlations among burnout dimensions and notable links between empowering and motivation. Overall network density was 33.3%. Mindfulness correlated negatively with devaluation (r = −0.24) and positively with empowering (r = 0.25). Empowering correlated positively with intrinsic motivation (r = 0.26), while disempowering correlated positively with extrinsic motivation. A motivation related positively to devaluation (r = 0.35), and extrinsic motivation correlated with reduced sense of accomplishment (r = 0.16).

**Figure 2:**
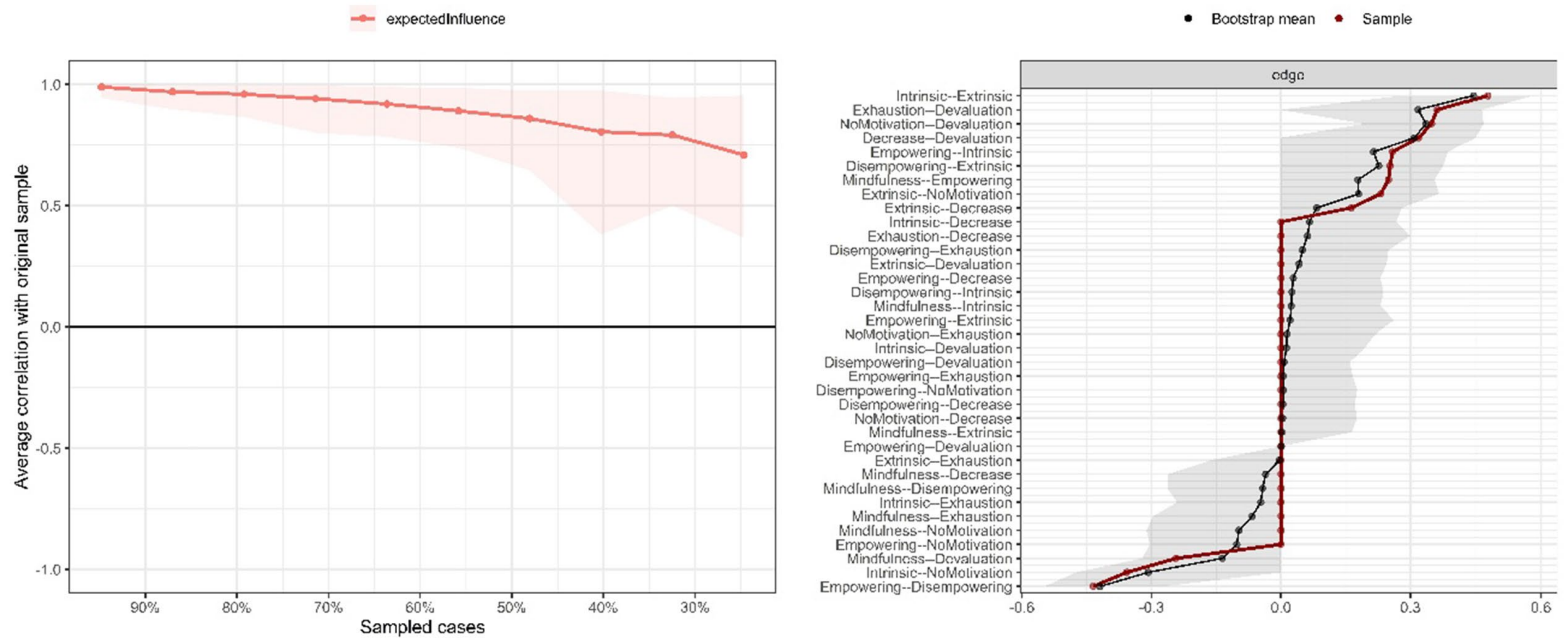
Network stability and accuracy analysis.

Regarding centrality, extrinsic motivation was the most influential node, followed by devaluation. In Figure 2, the 95% confidence intervals obtained through a non-parametric bootstrap with 1,000 resamples are presented, allowing for an assessment of the stability and precision of the relationships within the network. Overall, the narrow intervals and the fact that they do not include zero suggest consistent associations among the variables examined. Notably, the strongest connections such as those between intrinsic motivation and empowering climate, and between exhaustion and devaluation are also the most stable. In addition, the stability coefficient (CS = 0.44, CS > 0.25) supports the overall robustness of the model, indicating that the structure of the network remains largely unchanged even after 1,000 resamples.

## 4 Discussion

The findings deepen our understanding of psychological interactions emerging in virtual training among youth footballers an understudied yet highly relevant context post COVID-19. Descriptively, the sample showed high participation in remote activities, good adherence, and sustained coach follow-up, albeit with unequal access to training resources. In the absence of in-person contact, virtual communication became the primary medium for support, structure, and belonging (Hussain et al., 2023; Uroh and Adewunmi, 2021), making the quality of the coach-created climate pivotal.

However, it is important to note that these results reflect the specific conditions of a Peruvian professional academy operating during lockdown, and therefore should not be generalized to all football environments or sports disciplines without considering contextual, cultural, and technological differences.

### 4.1 Motivational climate and self-determined motivation

The network revealed a positive link between empowering climate and intrinsic motivation, and a direct association between disempowering climate and extrinsic motivation. These findings support SDT’s core tenet (Ryan and Deci, 2017): contexts that support autonomy, competence, and relatedness foster more self-determined regulation, whereas controlling environments foster externally regulated forms. During lockdown, coach mediation through virtual environments proved decisive. Prior evidence indicates that autonomy support in non-face-to-face settings can sustain involvement and enjoyment (Appleton and Duda, 2016; Castillo-Jiménez et al., 2022). Consistent with this, players perceiving empowering leadership reported higher intrinsic motivation and lower risk of emotional strain. Conversely, exposure to disempowering styles criticism, control, comparison was associated with extrinsic motivation and increased vulnerability to exhaustion, in line with Birr et al. (2023) and Martínez-González et al. (2025). Notably, extrinsic motivation emerged as the most central node, suggesting that under virtual conditions reliance on external feedback intensifies, thereby heightening emotional vulnerability.

From an applied perspective, these findings emphasise the importance of establishing clear pedagogical policies that formalise empowering communication in virtual training such as structured check-ins, autonomy supportive feedback, and performance goals tailored to athletes’ home contexts. However, implementing these policies demands addressing practical challenges common in football environments limited technology access, coach workload, and inconsistent institutional guidelines for remote monitoring barriers already reported among Latin American coaches (Álvarez, 2021) and further corroborated by global analyses of lockdown era sports training (Washif et al., 2022).

### 4.2 Mindfulness as a protective factor

Mindfulness correlated positively with empowering climate and negatively with sport devaluation, indicating a protective effect against psychological strain. This aligns with Li et al. (2019), who showed mindfulness moderates the stress–burnout relationship in high-performance athletes, and with Oguntuase and Sun (2022), who reported improvements in emotion regulation and resilience following brief mindfulness interventions in elite footballers. In the present study, mindfulness disposition appears to buffer the effects of virtuality and isolation by promoting present-moment awareness and reducing identification with evaluative or self-critical thoughts. From a network perspective, mindfulness acts as a modulating node, weakening connections among burnout dimensions especially valuable in remote settings where stress from social disconnection and uncertainty is heightened (O’Connor et al., 2022; Solmaz and Yarayan, 2025).

These results suggest that psychological departments and coaching staffs could integrate ultra-brief mindfulness routines (e.g., 3-min breath-focused practices, grounding exercises, or pre-session attentional resets) into virtual sessions. However, the implementation of such practices may face barriers such as insufficient psychological personnel, limited coach training in contemplative techniques, or resistance from athletes unfamiliar with mindfulness-based approaches. Recognizing these constraints is essential to designing feasible, scalable interventions adapted to resource-variable academy contexts.

### 4.3 Burnout and emotion regulation

Burnout dimensions exhaustion, devaluation, and reduced accomplishment were positively interrelated, consistent with Raedeke and Smith’s (2001) tridimensional model. In the network, they formed a tightly connected cluster, reflecting the syndrome’s progressive nature: physical/emotional exhaustion often precedes affective distancing and diminished efficacy. The observed association between extrinsic motivation and reduced accomplishment suggests that when sport behavior is sustained mainly by external incentives (e.g., coach approval, performance expectations), vulnerability to strain increases. Prior research already pointed to autonomy frustration and excessive control as robust burnout predictors (Madigan et al., 2023; Woods et al., 2022). During the pandemic, confinement and disrupted routines acted as risk multipliers (Hussain et al., 2023; Zhao et al., 2022). Our data reinforce viewing burnout not solely as an individual phenomenon but as the outcome of a systemic imbalance among contextual demands, motivation, and self-regulatory resources. In this sense, the pandemic functioned as a multiplier of psychological wear (Casas-Apayco et al., 2025), underscoring consistent with Brandão et al. (2024) the importance of attending to environmental and historical contexts and their impact on athletes’ psychology.

Importantly, these burnout patterns must be interpreted within the specific characteristics of this sample: adolescents from a single Peruvian academy, engaging in virtual training in reduced home spaces and under strict lockdown. These contextual factors limit the generalizability of the findings to other countries, age groups, competitive levels, or post-pandemic conditions where training demands, technological resources, and institutional structures differ substantially.

### 4.4 Practical implications

Findings offer applied implications for coach education and psychological intervention in virtual or hybrid sport contexts. First, coach training programs should emphasize empowering strategies such as autonomy support, constructive communication, and effort-based reinforcement which have demonstrated effectiveness in improving motivation, wellbeing, and persistence in youth athletes (Duda, 2013).

Experimental evidence also shows that structured workshops focused on need-supportive communication enhance athletes’ engagement, perceived competence, and emotional functioning (Cheon et al., 2012; Cheon and Reeve, 2013).

Second, micro-mindfulness interventions (3–5 min) can be embedded in virtual sessions to improve attentional control and emotional regulation. Brief mindfulness-based practices such as focused breathing, grounding techniques, and micro body scans have shown positive effects on stress reduction and recovery among athletes (Noetel et al., 2017). Manuals for applied sport psychology provide operational guidelines for designing and implementing such brief practices (Bühlmayer et al., 2017).

Finally, academies should implement virtual-ready wellbeing screenings using brief digital tools such as the Athlete Psychological Strain Questionnaire (APSQ), validated for monitoring mental health and early detection of exhaustion and demotivation (Rice et al., 2019). Screenings can be administered weekly or bi-weekly, incorporating automated alerts for high-risk indicators, consistent with best-practice models in early mental-health intervention.

Together, these strategies provide a concrete, feasible framework for supporting athletes’ psychological functioning during virtual or hybrid sport participation.

### 4.5 Limitations and future directions

The cross-sectional design precludes establishing causal relationships among motivational climate, motivation, mindfulness, and burnout. This design also limits temporal interpretation, as fluctuations in emotional states during prolonged lockdowns cannot be captured, and bidirectional effects (e.g., burnout influencing perception of climate) may remain obscured. The moderate sample size and single context setting further restrict generalizability. Specifically, a network estimated from a medium-sized sample may yield less stable estimates for weaker edges, and findings may reflect the unique socio-cultural, organizational, and technological characteristics of a single Peruvian professional academy operating exclusively in virtual mode during the pandemic. Therefore, caution is needed when extrapolating these results to other countries, training systems, sports, or post-pandemic conditions.

Future studies should adopt longitudinal or intervention designs to examine how motivational networks evolve over time or in response to targeted psychological programs. It will also be essential to incorporate contextual variables that may influence athletes’ psychological functioning during virtual training. Relevant variables include: (a) family support and parental involvement; (b) physical space available for training at home; (c) stability of internet connection and access to adequate devices; (d) perceived academic load; (e) emotional climate at home; and (f) availability of social interaction opportunities outside sport.

These variables could be integrated into future network models as additional nodes, moderators (in moderated network analysis), or hierarchical predictors through multilevel network modeling, allowing a more precise understanding of how environmental constraints shape psychological dynamics.

Hybrid models combining face-to-face sessions with digital psychological support should also be explored to evaluate how virtual interventions complement on-field practice and whether they buffer emotional strain in adolescent athletes.

## 5 Conclusion

Empowering motivational climates and mindfulness emerged as protective factors for the psychological functioning of adolescent footballers during virtual training, whereas disempowering leadership and extrinsic forms of motivation were associated with higher vulnerability to burnout. These findings highlight the importance of creating supportive and autonomy-enhancing environments, even when interaction is mediated through digital platforms.

However, the conclusions of this study should be interpreted with caution due to methodological and contextual specificities. The results derive from a single Peruvian academy, a restricted age group, a football-specific sample, and a pandemic-driven virtual training context, which limits generalization to other cultures, sports, gender compositions, or post-pandemic conditions. Future studies should examine whether similar psychological dynamics emerge in in-person settings, different sport modalities, or broader competitive levels.

Importantly, the proposed integrative framework connecting Empowering Coaching, Self-Determination Theory, and mindfulness offers a novel contribution by examining how these models interact simultaneously within a network structure. Unlike previous studies that analyzed these constructs in isolation, our network approach reveals their dynamic interdependence, identifying central and bridging variables that are not detectable through traditional linear analyses. This provides a methodological advancement and a more nuanced understanding of how motivation, attention, and emotional regulation interact under constraints such as virtuality.

Overall, the study underscores the relevance of designing psychologically sustainable training environments that consider autonomy support, attentional regulation, and emotional wellbeing not as isolated components, but as interconnected processes that shape athletes’ adaptation in both challenging and evolving sport contexts.

## Data Availability

The raw data supporting the conclusions of this article will be made available by the authors, without undue reservation.
